# Prolonged PR Interval and Outcome in Cardiac Resynchronization Therapy

**DOI:** 10.5935/abc.20190101

**Published:** 2019-07

**Authors:** Jakrin Kewcharoen, Chanavuth Kanitsoraphan

**Affiliations:** University of Hawaii Internal Medicine Residency Program, Honolulu, HI - USA

**Keywords:** Heart Failure, Cardiac resynchronization Therapy/methods, Meta-Analysis, Atrioventricular Block, Heart Conduction, System

**Dear Editor**

We read the article “Baseline Prolonged PR Interval and Outcome of Cardiac Resynchronization Therapy: A Systematic Review and Meta-analysis” by Rattanawong et al.^1^ with great interest. The authors investigated the effect of prolonged PR interval on composite outcome among patients who received cardio resynchronized therapy. The authors conclude that prolonged PR is associated with worse outcome in this population.

I would like to commend the authors for performing a systematic review and meta-analysis on this topic. The effect of prolonged PR interval was well-known in the general population. However, in this specific population, the answer is unclear and was in needed for answers, given almost half of the patients with cardiac resynchronization therapy (CRT) had 1^st^ degree AV block.^2^ Although several previous studies reported a negative impact on the cardiovascular outcome, they were retrospective cohort and was limited by a relatively small number of participants. Nevertheless, the authors demonstrated a good systematic review and performed the random-effect model with statistical perfection.

However, as the authors mentioned in the article, this meta-analysis has a limitation regarding the number of the included studies. Also, there is a difference in the cut-point for prolonged PR interval among the included studies. Although the study by Friedman et al. was the only study that used the cut-point of 230 ms instead of 200 ms, they have the most included participants and has the most weight percentage in all three forest plots. We thought that this is a crucial point as a change in risk ratio of the study by Freidman et al.^3^ could potentially change the overall pooled RR of the meta-analysis.

There are two additional studies that we thought could be included in the meta-analysis that could affect the outcome of this meta-analysis. Studies by Stockburger et al.^4^ and Lin et al.^5^ evaluated the effect of PR interval in patients with CRT.

Stockburger et al.^4^ performed a post hoc analysis from the Multicenter automatic defibrillator implantation trial with cardiac resynchronization therapy (MADIT-CRT) trial. The authors found that patients with CRT who had short PR interval (< 230 ms) generally had worse outcome regarding heart failure and all-cause mortality, regardless of the QRS duration, although the authors did not directly compare the prolonged PR interval to normal PR interval.

Another study by Lin et al.^5^ was based on the secondary analysis of The Comparison of Medical Therapy, Pacing, and Defibrillation in Heart Failure (COMPANION) trial. The authors reported that the outcome of all-cause mortality was reduced in both normal and prolonged PR interval group when compared to medical therapy alone, but more so in the prolonged PR interval group. Moreover, in a multivariable Cox model, progressively longer baseline PR interval, as a continuous variable, was associated with an increasingly greater reduction in the primary outcome.

Given that results from these two studies reported a positive correlation between PR interval and cardiovascular outcome, including the data from the two studies could potentially change the outcome of this meta-analysis.
