# Supplementary material for: Prolonged PR Interval and Outcome in Cardiac Resynchronization Therapy
Source: Arq Bras Cardiol. 2019 Jul;113(1):109–10. doi: 10.5935/abc.20190101 (PMC6684180; doi:10.5935/abc.20190101)
Supplement: Related texts

# Reply

**Authors:** Rattanawong Pattara, Prasitlumkum Narut, Riangwiwat Tanawan, Kanjanahattakij Napatt, Vutthikraivit Wasawat

We appreciate the author’s comments on our original article “Baseline Prolonged PR Interval and Outcome of Cardiac Resynchronization Therapy: A Systematic Review and Meta-analysis”.^1^ We agree with the authors that the effect of PR interval in cardiac resynchronization therapy (CRT) population is unknown, and therefore we conducted a systematic review and meta-analysis on this topic.

We agree that the study by Friedman et al.^2^ has the most included participants and has the most percentage in all three forest plots. However, to evaluate the effect of Friedman et al.,^2^ we performed a sensitivity analysis by excluding one study at a time which is shown in Supplementary Document 2 in the original article.^1^ After excluding Friedman et al.,^2^ there was no significant change of the risk ratio of composite outcome (risk ratio = 1.19, 95% confidence interval: 1.04-1.36), all-cause mortality (risk ratio=1.53, 95% confidence interval: 1.23-1.91), and heart failure hospitalization (risk ratio = 1.6, 95% confidence interval: 1.06-2.42).^1^ The meta-analysis pooled result still has a statistically significant risk ratio even if we exclude the study by Friedman et al.^2^ Therefore, a prolonged PR interval still predicts the outcomes of CRT regardless of the results of the study by Friedman et al.^2^

We appreciate the author’s recommendation to include the study by Stockburger et al.^3^ and Lin et al.^4^ Both studies were included from our comprehensive search and both full-text articles were reviewed. However, both studies were excluded from our systematic review and meta-analysis for the following reasons.

The study by Lin et al.^4^ was excluded from our systematic review and meta-analysis due to an inappropriate comparison as demonstrated in Figure 1 of our original article.^1^ Their objective is to compare the outcomes between CRT and optimal pharmacologic therapy stratified by PR interval. Our meta-analysis compared the outcome of prolonged PR interval versus normal PR interval only in CRT patients.

Similarly, the study by Stockburger et al.^3^ was also excluded from our systematic review and meta-analysis due to an inappropriate comparator. Stockburger et al.^3^ reported that patients with prolonged PR interval derived clinical benefit with reduction in heart failure or death from CRT when compared to an implantable cardioverter defibrillator.^3^ Therefore, their comparison was different from our study, which was prolonged PR interval versus normal PR interval in CRT patients.

Our systematic review and meta-analysis used comprehensive search methodology and strict inclusion and exclusion criteria; therefore our results are robust. We showed that a prolonged PR interval in CRT patients is significantly associated with an increased risk for all-cause mortality, composite outcome, and HF hospitalization when compared to normal PR interval in CRT patients.^1^

## References

[1] Rattanawong P, Prasitlumkum N, Riangwiwat T, kanjanahattaki N, Vuthikraivit W, Chongsathidkiat P (2018). Baseline Prolonged PR Interval and Outcome of Cardiac
Resynchronization Therapy: A Systematic Review and
Meta-analysis. Arq Bras Cardiol.

[2] Friedman DJ, Bao H, Spatz ES, Curtis JP, Daubert JP, Al-Khatib SM (2016). Association between a prolonged PR interval and outcomes of
cardiac resynchronization therapy: a report from the National
cardiovascular Data-Registry. Circulation.

[3] Stockburger M, Moss AJ, Klein HU, Zareba W, Goldenberg I, Biton Y (2016). Sustained clinical benefit of cardiac resynchronization
therapy in non-LBBB patients with prolonged PR-interval: MADIT-CRT
long-term follow-up. Clin Res Cardiol.

[4] Lin J, Buhr KA, Kipp R (2017). Effect of PR Interval on Outcomes Following Cardiac
Resynchronization Therapy: A Secondary Analysis of the COMPANION
Trial. J Cardiovasc Electrophysiol.
